# Prostate cancer small non-coding RNA transcriptome in Arabs

**DOI:** 10.1186/s12967-017-1362-x

**Published:** 2017-12-21

**Authors:** Jingxuan Shan, Khalid Al-Rumaihi, Karim Chouchane, Issam Al-Bozom, Danny Rabah, Karim Farhat, Lotfi Chouchane

**Affiliations:** 1 Laboratory of Genetic Medicine and Immunology, Weill Cornell Medicine-Qatar, Education City, Qatar Foundation, Doha, Qatar; 2 Department of Urology, Hamad Medical Corporation, Doha, Qatar; 3 Faculty of Medicine and Surgery, Universita Cattolica del Sacro Cuore, Rome, Italy; 4 Department of Laboratory Medicine and Pathology, Hamad Medical Corporation, Doha, Qatar; 5 Department of Surgery, Cancer Research Chair, College of Medicine, King Saud University, Riyadh, Saudi Arabia

**Keywords:** Prostate cancer, Small RNA transcriptome, miRNA, miRNA editing, Arabs

## Abstract

**Background:**

Prostate cancer (PCa) is a complex disorder resulting from the combined effects of multiple environmental and genetic factors. Small non-coding RNAs (sRNAs), particularly microRNAs (miRNAs), regulate several cellular processes and have an important role in many human malignancies including PCa. We assessed the sRNA profiles associated with PCa in Arabs, a population that has rarely been studied.

**Methods:**

We used next generation sequencing technology to obtain the entire sRNA transcriptome of primary prostate tumor formalin-fixed paraffin-embedded tissues, and their paired non-tumor tissues, collected from Bedouin patients (Qatari and Saudi). The miRNA and the target gene expression were evaluated by real-time quantitative PCR. miRNA KEGG pathway and miRNA target genes were subsequently analyzed by starBase and TargetScan software.

**Results:**

Different expression patterns of several sRNA and miRNA editing were revealed between PCa tumor and their paired non-tumor tissues. Our study identified four miRNAs that are strongly associated with prostate cancer, which have not been reported previously. Differentially expressed miRNAs significantly affect various biological pathways, such as cell cycle, endocytosis, adherence junction and pathways involved in cancer. Prediction of potential targets for the identified miRNAs indicates the overexpression of KRAS, BCL2 and down-regulation of PTEN in PCa tumor tissues.

**Conclusion:**

These miRNAs, newly associated with prostate cancer, may represent not only markers for the increased risk of PCa in Arabs, but may also reflect the clinical and pathological diversity as well as the ethno-specific heterogeneity of prostate cancer.

**Electronic supplementary material:**

The online version of this article (10.1186/s12967-017-1362-x) contains supplementary material, which is available to authorized users.

## Background

Prostate cancer is the most common malignancy in Western countries and the second cause of cancer-related death in Europe and the United States [1]. With lifestyle changes, the incidence of the disease has been increasing in the Arab populations [2]. From 1991 to 2006, PCa was the most common cancer in Qatari males over 65 years old [3]. In Kuwait, the incidence of prostate cancer rose to 12.3/100,000 men/year in 2004 [4]. In Arab populations, the incidence of PCa correlates with a low prostate volume and a low testosterone level. The high frequency of aggressive forms of PCa in Arab patients, despite the low levels of testosterone, indicates an increased sensitivity of Arab men to this steroid [5].

Prostate cancer is generally considered a complex disease and several genes underlie its onset, course, and severity. The genetic susceptibility to prostate cancer is variable among different populations [6]. The identification of population-specific genetic variants may help to better understand the genetics and the molecular mechanisms of prostate cancer.

At present, PCa is diagnosed primarily through the use of digital rectal examination and the measurement of serum levels of prostate-specific antigen (PSA). However, PSA is not prostate cancer specific and can be found with normal prostate at equal or higher levels than in PCa. The non-specificity of PSA was particularly reported for Middle-Eastern and North African populations [7]. The poor specificity of serum PSA, the only current biomarker of the disease, presents significant problems for disease diagnosis, patient treatment and management. It is widely admitted that more specific prognostic and diagnostic markers of PCa are urgently needed.

Next generation sequencing (NGS) studies have revealed that the majority of the human genome is transcribed, with thousands of non-protein-coding RNAs (ncRNA), which comprise small and long ncRNAs [8, 9]. Alterations in the expression of miRNA genes, which are small RNAs having 19–25 base pairs (bp) in length, contribute to the pathogenesis of most, perhaps all, human malignancies [10–12]. Several findings support an important role of the small non-coding RNAs in PCa [13–18]. Studies of PCa-specific miRNAs show potential for their utilization in the diagnosis and treatment of PCa [13–15]. Moreover, ribosomal RNA (rRNA) modification, small nuclear RNAs (snRNAs) and small nucleolar RNAs (snoRNAs) have been shown to be involved in PCa progression [16–18]. Previous studies, which have assessed the small RNA transcriptome in PCa and/or in different subtypes of PCa, are summarized in [15]. Most, if not all, PCa sRNA data, including miRNAs obtained so far, originated from Western and Asian specimens, and significant differences in prostate tumor pathological and clinical characteristics have been found between different ethnicities [19, 20].

With the aim to identify an sRNA signature associated with prostate cancer in Arabs, we first conducted a deep sequencing of the entire small RNA transcriptome in PCa tissues along with non-malignant adjacent tissues. We further extended the study to validate the expression of several miRNAs and to search for potential targets associated with their deregulation in prostate cancer.

## Methods

### Patients and sample collection

Thirty-two patients with prostate cancer from Qatar and Saudi Arabia, from Bedouin tribes, were included in this study. Informed consents were obtained from all patients, and the study protocol was approved by the Institutional Review Boards of Weill Cornell Medicine-Qatar, Hamad Medical Corporation and King Saud University Hospital. The age and Gleason score of Qatari (Q) and Saudi (S) patients are listed in Additional file 1: Table S1. All the tissues collected from prostate cancer surgical specimens and the FFPE prostate tissues were stored in Hamad Medical Corporation and in King Saud University Hospital.

The areas of tumor and normal tissue sampling were identified by pathologists, and 3 sections of 10 μm in the thickness of each FFPE tissue were taken for RNA extraction. Total RNA was extracted with RecoverAll Total Nucleic Acid Isolation Kit (Ambion, USA) following manufacturer’s protocols. The quantity and quality of RNA were examined by Agilent 2100 Bioanalyzer (Agilent Technologies, USA).

### Small RNA transcriptome sequencing

Next generation sequencing (NGS) technology was used to obtain the entire sRNA transcriptome of 20 samples (10 primary prostate tumor FFPE tissues, and their paired non-tumor tissues). Briefly, small RNAs in the size range from 18 to 30 nt were gel purified and ligated to 5′ and 3′ adaptor, and the ligation products were subjected to reverse transcription and then amplified for 15 cycles using the adaptor primers. The fragments around 150 bp were isolated and sequenced on Illumina HiSeq 2000 platform (Illumina, USA).

### NGS data analysis

Raw reads went through data cleaning first, which includes removing adaptors, getting rid of low quality tags and several kinds of contaminants from the 50 nt tags. Length distribution of clean tags was then summarized. Clean reads were mapped to genome hg18 track by Short Oligonucleotide Analysis Package (SOAP) to analyze their expression and distribution.

To obtain the miRNA expression profile, small RNA tags were aligned to the precursor/mature miRNA of Homo Sapiens in miRBase18. Small non-coding RNA tags with rRNA, snRNA, snoRNA, small cytoplasmic RNA (scRNA) and transfer RNA (tRNA) were annotated in Genbank and Rfam. After excluding all the matched tags, the remaining sequencing reads were aligned to exons and introns of mRNA to identify the degraded fragments of mRNA. All the unannotated small RNA tags might represent novel miRNA and base edits of potential known miRNA.

The comparisons of percentage between tumor and normal tissues were calculated using paired one-tailed *t* test.

### Real time-quantitative PCR (RT-qPCR)

For mRNA expression, total RNA was reverse transcribed into cDNA using oligo 16T primer and then gene expression was relatively quantified with GoTaq^®^ 2-Step RT-qPCR System for SYBR Green-based detection on Applied Biosystems^®^ 7500 fast real-time PCR machine. The HPRT1 gene was used as a reference. The sequences of primers are listed in Additional file 1: Table S2.

For miRNA expression, total RNA was reverse transcribed using miRNA specific primer with TaqMan MicroRNA Reverse Transcription Kit (Applied Biosystems, USA). The miRNA levels were quantified with Taqman probe-based detection (Applied Biosystems, USA) on Applied Biosystems^®^ 7500 fast Real-Time PCR Machine. The 18s rRNA was used as a reference.

## Results

### Small non-coding RNA transcriptomes of Arab prostate cancer specimens

Small RNA transcriptomes from a total of 10 pairs of FFPE PCa tissues and their adjacent normal tissues were analyzed by NGS. A total of 766,824,250 high quality reads were obtained from the sequencing. After removal of irrelevant sequences there were 691,235,882 total reads. The length distribution analysis revealed that the RNA sequences were mainly within a range of 20–23 nt (Additional file 1: Figure S1), which corresponds to the size of most known small RNAs.

### Library composition and mapping results

**Fig. 1:**
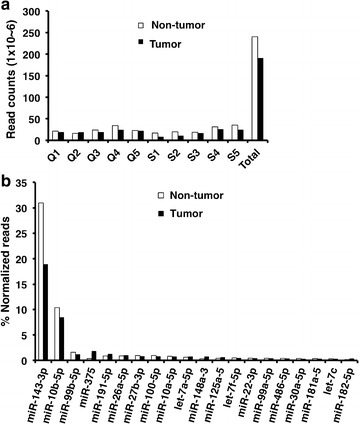
Mapping results of small RNA transcriptome of 10 pairs of PCa tumor and non-tumor tissues. **a** Mapped small RNA total read counts in each specimen and the cumulative total reads counts in tumors and non-tumor tissues. **b** Top 10 known miRNAs expressed in tumor and non-tumor tissue detected by NGS. Calculated percentage of each miRNA to total known miRNA reads is presented

For each sample, 19 to 38 million reads were mapped to the human genome. For all samples, the percentage of alignments exceeded 70% (Additional file 1: Table S3). These reads included miRNAs, rRNAs, tRNAs, scRNAs, snRNAs, snoRNAs, sRNA repeats, exons, introns, and unknown nucleotide sequences (Table 1). In most of the cases, the total mapped reads were higher in non-tumor tissues than in tumor tissues (Fig. 1a). The read count percentages (Table 1) for snRNA, snoRNA, scRNA and sRNA repeats were significantly higher in PCa tumor tissues than in non-tumor tissues (*P* = 0.015; *P* = 0.002; *P* = 0.049 and *P* = 0.01 respectively). Conversely, the read count percentage for miRNA was significantly lower in PCa tumor tissues (*P* = 0.024).

**Table 1:** Small RNAs in 10 pairs of FFPE prostate cancer specimens detected by NGS

Sample	Total	Exon antisense	Exon sense	Intron antisense	Intron sense	miRNA	rRNA	Repeat	scRNA	snRNA	snoRNA	tRNA	Unann
Q1N	28,277,250^a^ 100	2403 0.01	179469 0.63	6658 0.02	19,589 0.07	21,189,795 74.94	1,066,129 3.77	18,421 0.07	20,980 0.07	29,803 0.11	57,285 0.20	326,441 1.15	5,360,132 18.96
Q1T	27,918,838 100	3281 0.01	189,423 0.68	9349 0.03	21,878 0.08	19,224,820 68.86	1,327,029 4.75	23,952 0.09	88,860 0.32	82,114 0.29	203,246 0.73	1,064,063 3.81	5,680,444 20.35
Q2N	35,391,692 100	3470 0.01	298,030 0.84	26,105 0.07	80,756 0.23	15,998,067 45.2	5,369,926 15.17	76,891 0.22	196,764 0.56	199,951 0.56	221,878 0.63	8,445,871 23.86	4,472,358 12.64
Q2T	33,773,903 100	10,013 0.03	524,796 1.55	25,779 0.08	164,968 0.49	18,912,932 56.00	4,493,690 13.31	158,765 0.47	106,115 0.31	855,596 2.53	690,244 2.04	767,289 2.27	7,062,748 20.91
Q3N	35,735,732 100	1705 0.00	297,385 0.83	4644 0.01	24,114 0.07	23,955,712 67.04	1,974,819 5.53	35,437 0.10	151,635 0.42	71,211 0.20	131,070 0.37	1,937,351 5.42	7,150,228 20.01
Q3T	36,095,235 100	4090 0.01	497,973 1.38	8652 0.02	82,083 0.23	19,275,478 53.40	3,251,981 9.01	104,960 0.29	189,016 0.52	281,679 0.78	516,959 1.43	5,106,698 14.15	6,774,339 18.77
Q4N	45,235,749 100	2750 0.01	68,350 0.15	5387 0.01	14,524 0.03	34,012,084 75.19	1,382,848 3.06	29,433 0.07	71,732 0.16	30,671 0.07	71,326 0.16	982,107 2.17	8,564,307 18.93
Q4T	32,598,915 100	2939 0.01	52,485 0.16	8446 0.03	18,961 0.06	24,364,229 74.74	690,166 2.12	45,388 0.14	43,589 0.13	32,698 0.10	106,106 0.33	484,692 1.49	6,749,107 20.70
Q5N	34,197,138 100	15,613 0.05	1,110,918 3.25	4628 0.01	19,319 0.06	22,754,694 66.54	1,418,810 4.15	25,247 0.07	31,011 0.09	32,193 0.09	54,705 0.16	307,863 0.90	8,421,998 24.63
Q5T	35,779,358 100	8384 0.02	297,959 0.83	8508 0.02	30,761 0.09	21,985,915 61.45	2,656,228 7.42	49,736 0.14	116,160 0.32	167,169 0.47	270,220 0.76	1,596,387 4.46	8,590,786 24.01
S1N	32,055,346 100	6117 0.02	1,845,217 5.76	12,057 0.04	60,258 0.19	17,015,120 53.08	6,283,167 19.60	68,725 0.21	24,960 0.08	104,261 0.33	234,082 0.73	350,428 1.09	6,050,477 18.88
S1T	41,112,010 100	17,603 0.04	3,936,180 9.57	44,451 0.11	251,699 0.61	8,314,806 20.22	20,102,292 48.90	344,943 0.84	69,247 0.17	428,991 1.04	986,772 2.40	672,186 1.64	5,940,943 14.45
S2N	29,397,073 100	3898 0.01	897,203 3.05	7963 0.03	46,597 0.16	19,689,931 66.98	3,052,566 10.38	62,226 0.21	16,824 0.06	56,902 0.19	108,604 0.37	186,500 0.63	5,267,657 17.92
S2T	26,137,643 100	13,962 0.05	295,974 1.13	16,398 0.06	95,037 0.36	10,893,729 41.68	7,311,047 27.97	129,995 0.50	61,762 0.24	212,641 0.81	494,207 1.89	912,770 3.49	5,699,007 21.80
S3N	27,264,478 100	4766 0.02	226,353 0.83	15,660 0.06	39,350 0.14	18,702,182 68.60	2,369,035 8.69	92,587 0.34	30,752 0.11	57,087 0.21	127,926 0.47	230,624 0.85	5,367,833 19.69
S3T	30,690,489 100	9707 0.03	2,599,251 8.47	17,755 0.06	99,710 0.32	16,952,101 55.24	4,516,427 14.72	116,291 0.38	44,387 0.14	183,781 0.60	415,388 1.35	349,767 1.14	5,385,487 17.55
S4N	42,147,101 100	11,918 0.03	333,573 0.79	7982 0.02	43,044 0.10	31,240,514 74.12	3,129,257 7.42	52,155 0.12	30,886 0.07	64,665 0.15	129,612 0.31	273,707 0.65	6,829,490 16.20
S4T	34,881,814 100	6136 0.02	71,254 0.20	11,995 0.03	24,355 0.07	25,685,322 73.64	1,061,270 3.04	61,568 0.18	51,806 0.15	27,056 0.08	59,345 0.17	476,327 1.37	7,345,173 21.06
S5N	47,111,742 100	2764 0.01	495,709 1.05	11,953 0.03	31,017 0.07	35,338,827 75.01	1,482,687 3.15	61,971 0.13	70,137 0.15	56,524 0.12	127,766 0.27	462,461 0.98	8,969,539 19.04
S5T	35,434,376 100	4534 0.01	254,597 0.72	10,168 0.03	39,678 0.11	24,840,095 70.10	3,051,937 8.61	65,451 0.18	45,425 0.13	41,078 0.12	107,746 0.30	978,442 2.76	5,994,715 16.92

*Q* Qatar, *S* Saudi Arabia, *N* non-tumor, *T* tumor, *Unann* unannotated

^a^Reads/percentage

Up to 1311 miRNAs were detected from all samples, with a large dynamic of read counts ranging from 1 to 215,035,382 (Additional file 2: Table S4). Out of the 1311 miRNAs, 590 miRNAs have at least one count in more than 50% of samples, and only 247 miRNAs have an average more than 100 reads per sample (Additional file 2: Table S4). Expression comparison of the 247 miRNAs in PCa tissues and in their corresponding adjacent non-tumor tissues ranked miR-143-3p and miR-10b as the most abundant miRNAs in the PCa tumor tissues (about 50 and 20% of total miRNA reads, respectively). The expression of the top-ranked 20 miRNAs, representing more than 90% of the total miRNA reads in PCa tumor tissues, is shown in Fig. 1b.

### Prostate cancer miRNA expression profiling

**Fig. 2:**
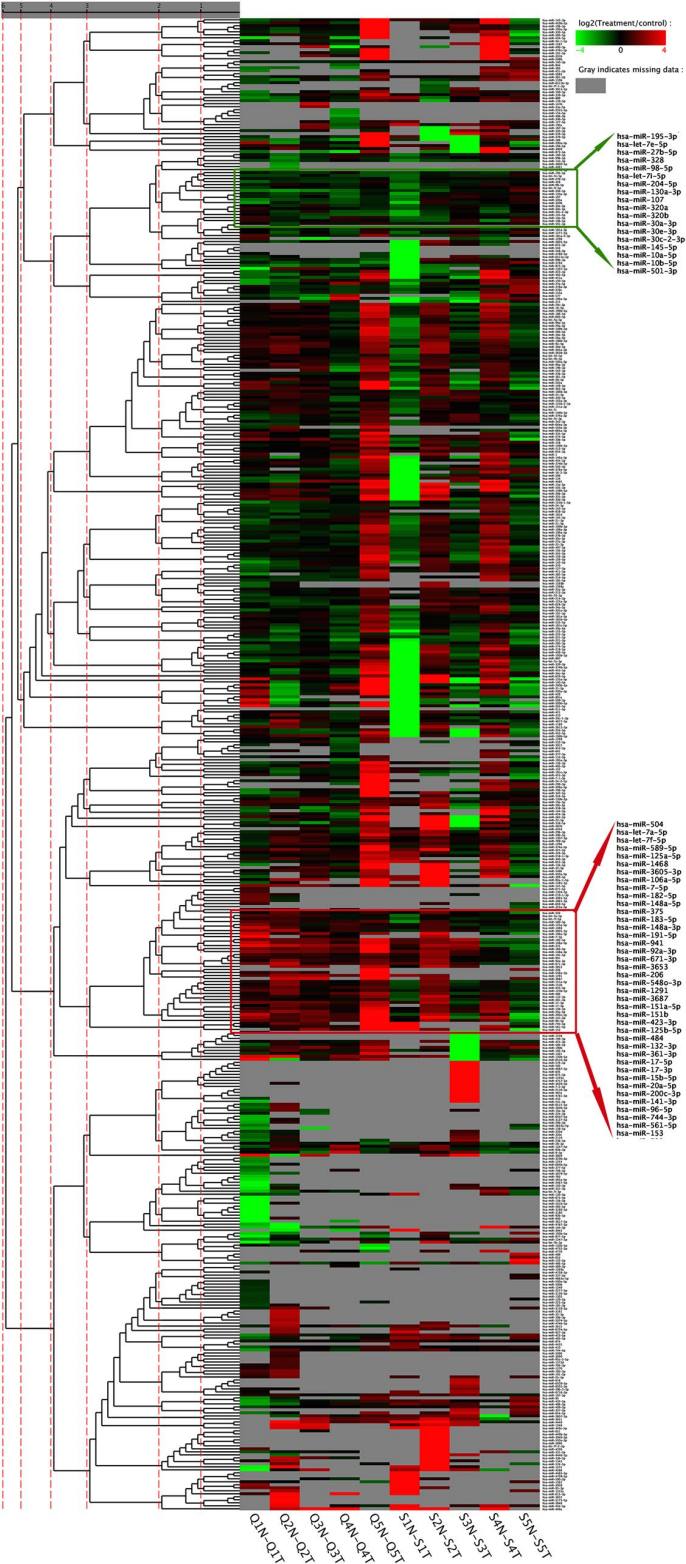
Cluster analysis of differentially expressed miRNAs in 10 pairs of PCa tumor and non-tumor tissues. Each row shows one miRNA and each column shows one sample pair. Therefore, each cell shows the differential expression of a miRNA in one sample pair. Red indicates that the miRNA has a higher expression in tumor tissue, green indicates that the miRNA has a higher expression in non-tumor tissue, and grey indicates that the miRNA has no expression (detected tag counts < 5) in at least one of the sample pair. miRNA with similar expression pattern in different sample pairs are clustered together

To compare the miRNA expression between tumor and non-tumor tissues, the actual miRNA counts were normalized into transcripts per million (TPM). The fold-change and *P*-value from the normalized expression were calculated. The results of each pair of samples are listed in Additional file 3: Table S5 and Additional file 1: Figure S2. The miRNA expression of all the 10 pairs of samples was subjected to an unsupervised cluster analysis (Fig. 2). All miRNAs with average reads below 100 per sample were filtered out. The cluster highlighted in red corresponds to a group of miRNAs upregulated in the PCa tumor tissues, whereas the one highlighted in green corresponds to those down-regulated (green box in Fig. 2). Twenty-seven miRNAs were upregulated and 18 downregulated in the PCa tumors (Table 2). Out of these 45 miRNAs, 26 have a read counts exceeding an average of 5000 per sample.

**Table 2:** Differentially expressed miRNAs (Tumor/non-tumor)

Up			Down		
miRNA	References	Note	miRNA	References	Note
hsa-let-7a-5p	[8]		hsa-let-7e-5p	[8]	
hsa-miR-125a-5p	[8]		hsa-miR-107	[8]	Up
hsa-miR-125b-5p	[8]		hsa-miR-130a-3p	[8]	
hsa-miR-141-3p	[8]		hsa-miR-143-3p	[8]	
hsa-miR-141-5p	[15]		hsa-miR-143-5p		
hsa-miR-148a-3p	[8]		hsa-miR-145-3p		
hsa-miR-148a-5p	[16]		hsa-miR-145-5p	[8]	
hsa-miR-148b-3p	[8]		hsa-miR-184	[8]	
hsa-miR-148b-5p	[17]		hsa-miR-195-3p		
hsa-miR-151a-5p	[8]		hsa-miR-204-5p	[8]	
hsa-miR-15b-5p	[8]		hsa-miR-221-3p	[8]	
hsa-miR-17-5p	[8]		hsa-miR-221-5p	[8]	
hsa-miR-182-5p	[8]		hsa-miR-24-3p	[8]	
hsa-miR-183-5p	[8]		hsa-miR-30a-3p	[8]	
hsa-miR-191-5p	[8]		hsa-miR-320a	[18]	
hsa-miR-200c-3p	[8]		hsa-miR-320b		
hsa-miR-20b-5p	[8]		hsa-miR-328	[8]	
hsa-miR-25-3p	[8]		hsa-miR-451a	[8]	
hsa-miR-363-3p	[8]				
hsa-miR-375	[8]				
hsa-miR-423-3p	[8]				
hsa-miR-425-5p	[8]				
hsa-miR-484	[8]				
hsa-miR-671-3p					
hsa-miR-92a-3p	[8]				
hsa-miR-93-5p	[8]				
hsa-miR-96-5p	[8]				

**Fig. 3:**
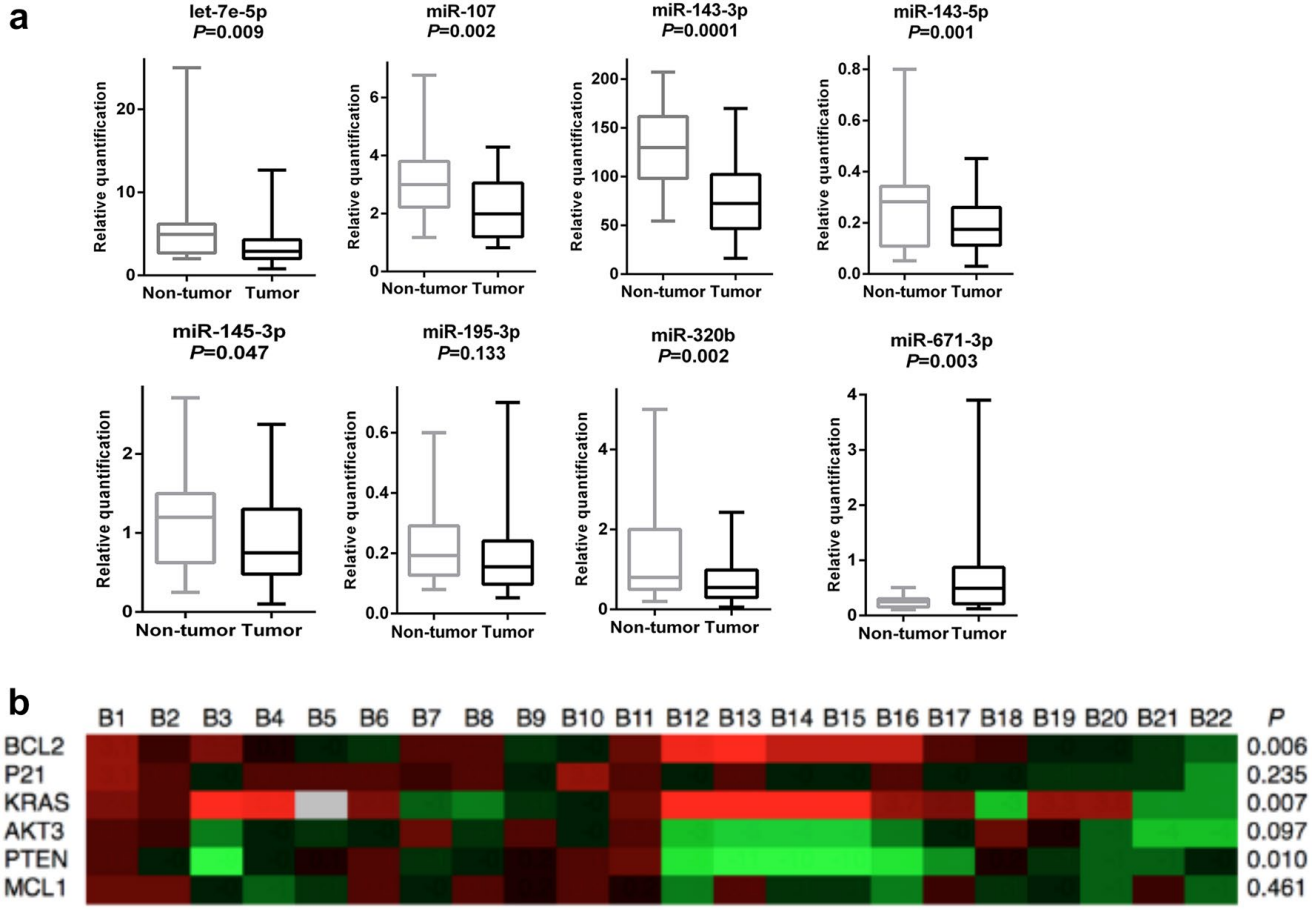
Validation of **a** miRNA and **b** mRNA expression profiles in additional 22 pairs of PCa tumor and non-tumor tissues of Bedouin patients. Data obtained by RT-qPCR. miRNA expression is normalized by RNU6B snRNA and mRNA expression is normalized by HPRT1. Expression comparisons between tumor and non-tumor tissues were calculated by paired two-tailed *t* test.** a** Relative quantification = 2^−Δ(CtmiRNA − CtRNU6B)^.** b** Each cell shows the differential expression of the mRNA in one sample pair. Red indicates that the mRNA has a higher expression in tumor tissue, green indicates that the mRNA has a higher expression in non-tumor tissue, and grey indicates that the mRNA has no expression in at least one of the sample pair

In contrast to the findings of a previous report [15], miR-107 was found to be down-regulated in PCa tumor tissues. Five miRNAs are newly found to be associated with PCa namely, miR-671-3p, miR-143-5p, miR-145-3p, miR-195-3p and miR-320b. Except for miR-671-3p, all other miRNAs were found down-regulated in tumor tissues. Validation of NGS findings was performed using RT-qPCR. As shown in Fig. 3a, except for miR-195-3p, NGS findings were replicated by RT-qPCR. Taken together, our results unveil four novel associations between miRNAs and PCa.

### Identification of miRNAs targets

Since the primary function of miRNA is to target mRNAs and interfere with their expression, we analyzed the KEGG pathways affected by the 45 miRNAs that were differentially expressed in PCa tumor tissues. The starBase tool, [21] based on microRNA-mRNA interactions from Argonaute CLIP-Seq and Degradome-Seq data, was applied. Pathways targeted by up-regulated miRNAs were expected to be negatively affected, whereas those targeted by down-regulated miRNAs would be over-expressed (Table 3). The KEGG ID: hsa05200 cancer pathway was found to be the most significant affected pathway. Both upregulated and downregulated miRNAs significantly affect cell cycle, endocytosis, and adherence junction. The prostate cancer pathway was found to be upregulated.

**Table 3:** miRNA-mRNA interaction targeted pathways in tumors (*P* < 0.001)

	KEGG ID	KEGG term	Hypergeometric *P*-value	Corrected *P*-value (BF)
	hsa05200	Pathways in cancer	6.16E−08	1.23E−05
	hsa04110	Cell cycle	7.96E−08	1.58E−05
	hsa04144	Endocytosis	1.34E−07	2.67E−05
Down	hsa04520	Adherens junction	2.55E−07	5.08E−05
	hsa04310	Wnt signaling pathway	1.16E−06	2.31E−04
	hsa05220	Chronic myeloid leukemia	4.39E−06	8.73E−04
	hsa04114	Oocyte meiosis	4.90E−06	9.75E−04
	hsa05200	Pathways in cancer	2.47E−09	5.14E−07
	hsa05220	Chronic myeloid leukemia	4.54E−08	9.45E−06
	hsa04144	Endocytosis	3.80E−07	7.91E−05
	hsa05215	Prostate cancer	7.45E−07	1.55E−04
Up	hsa04110	Cell cycle	7.71E−07	1.60E−04
	hsa04115	p53 signaling pathway	2.31E−06	4.80E−04
	hsa04120	Ubiquitin mediated proteolysis	2.33E−06	4.84E−04
	hsa05210	Colorectal cancer	2.51E−06	5.22E−04
	hsa04520	Adherens junction	2.82E−06	5.87E−04

*BF* Bayes-factors

Based on the miRNA target prediction results, we selected the top 6 most frequently targeted genes, relevant to cancer, and assessed their expression in 22 pairs of prostate tumor specimens and in their adjacent non-tumor tissues. KRAS and BCL2 oncogenes were highly expressed in tumor tissues, whereas the tumor suppressor PTEN gene was significantly downregulated (Fig. 3b).

### miRNA editing analysis

Transcriptome analysis is based commonly on the analysis of transcript levels and biological pathway alterations. Recently, more emphasis is placed on post-transcriptional modifications, particularly on RNA editing. This process targets not only mRNAs, but also small RNAs, including miRNAs. Adenosine to inosine (A-to-I) substitution, equivalent to A-to-G cDNA changes, is the most prevalent alteration. A-to-I changes in seed sequence (+ 2 to + 8 positions of mature miRNA) could modulate miRNA-binding specificity [22], and could modulate the maturation [23] and expression [24] in non-seed region. To get insight into miRNA editing in PCa, un-annotated sRNA tags, that align to mature miRNA with one base mismatch, were analyzed. A summary of read counts of edited and wild-type miRNAs are listed in Table 4. The obtained results indicate that for several miRNAs, the edited format predominates the miRNA pool. For certain miRNAs, such as miR-23c, editing could be seen in 100% of miRNA pool (Additional file 4: Table S6). miRNA editing is more frequent in PCa tumor tissues than in non-tumor tissues (*P* = 0.0560). Positive correlation between miRNA editing and miRNA expression pattern was seen only for let-7e-5p miRNA (Table 5).

**Table 4:** Statistics of miRNA editing in PCa samples

Patient	N/T	Count with base edit	Count without base edit	Count with base edit/total (%)
Q1	N	5,321,896	21,198,776	25.10
	T	7,787,197	19,220,573	28.83
Q2	N	5,823,249	15,969,875	26.72
	T	7,467,449	18,853,718	28.37
Q3	N	8,579,821	23,983,810	26.35
	T	9,210,644	19,298,828	32.30
Q4	N	8,076,872	34,118,648	19.14
	T	7,532,111	24,404,950	23.58
Q5	N	6,186,388	22,761,105	21.37
	T	8,750,023	22,044,971	28.41
S1	N	10,050,073	17,020,307	37.13
	T	4,820,530	8,264,552	36.83
S2	N	8,645,936	19,689,897	30.51
	T	5,213,543	10,899,980	32.36
S3	N	6,020,810	18,710,729	24.34
	T	7,471,140	16,950,204	30.59
S4	N	12,781,917	31,251,537	29.03
	T	6,021,975	25,742,476	18.96
S5	N	11,084,722	35,390,547	23.85
	T	13,259,479	24,854,309	34.79
Total	N	82,571,684	240,095,231	25.59
	T	77,534,091	190,534,561	28.92

*Q* Qatar, *S* Saudi Arabia, *N* non-tumor, *T* tumor

**Table 5:** Statistics of hsa-let-7e-5p editing

Patient	Non-tumor			Tumor		
	Edited	Wildtype	Percentage	Edited	Wildtype	Percentage
Q1	240,370	87,935	73.22	557,205	44,596	92.59
Q2	446,002	64,943	87.29	854,634	32,478	96.34
Q3	212,470	9154	95.87	354,812	8077	97.77
Q4	151,335	8809	94.50	92,280	3395	96.45
Q5	116,611	17,170	87.17	142,264	7492	95.00
S1	535,538	27,674	95.09	197,120	11,975	94.27
S2	298,654	21,904	93.17	296,729	16,490	94.74
S3	174,168	20,140	89.64	203,879	15,356	93.00
S4	115,859	8238	93.36	83,231	4713	94.64
S5	235,026	12,658	94.89	194,898	14,228	93.20

*Q* Qatar, *S* Saudi Arabia

## Discussion

Significant data on small RNA profiling in prostate cancer has been accumulated from population studies of different ancestries, including Europeans and Asians. Arab populations, including Arab Gulf populations, however, have not been studied. To our knowledge, this is the first study to unveil small RNA profiles associated with prostate cancer in Arab populations, in which aggressive forms of prostate cancer are frequently found.

Our analysis of the entire small non-coding RNA profile of prostate tumors collected from Arab patients led to more than 691 million clean reads. Since miRNA reads account for more than 70% of all the small RNA reads, we focused our analysis on miRNAs. We found that 45 miRNAs were significantly deregulated in PCa tumor tissues. We specifically identified the KEGG pathways targeted by these deregulated miRNAs. We further assessed the expression levels of oncogene and tumor suppressor genes most frequently targeted by these deregulated miRNAs.

Our findings are consistent with several reports (summarized in [15]), which showed positive association of several miRNAs with prostate cancer. However, our study unveiled novel associations in Arab patients. We report here 4 miRNAs, which are associated with prostate cancer for the first time, namely miR-671-3p, miR-143-5p, miR-145-3p and miR-320b. Our findings, along with the report indicating a significant association of miR-671-3p with breast cancer [25], suggest that miR-671-3p could be an attractive marker for prostate cancer risk.

Using in silico analysis, the search for potential targets of the miRNAs associated with prostate cancer showed that the KEGG ID: hsa05200 cancer pathway is the most significantly affected pathway. Gene expression quantification of selected oncogenes and tumor suppressor genes, involved in this pathway showed that KRAS and BCL2 were consistently upregulated in prostate tumor specimen, whereas PTEN was consistently downregulated. No significant changes were seen in the expression of P21, AKT3 and MCL1. This result suggests that miRNAs do not play a major role in the regulation of the expression of these genes. While miRNA deregulation has been associated with aberrant expression of BCL2, KRAS and PTEN in other type of cancers [26–29], further studies are needed to shed light on their role in prostate cancer.

Our analysis indicates that the scRNA, snRNA, snoRNA and repeat sRNA are over-expressed in prostate cancer tissues. This could make cancer cells more versatile and more responsive to environmental changes. Conversely, and as previously reported [30], the total miRNA reads in prostate tumor tissues were found to be lower compared to that found in non-tumor tissues. Several studies of various tumors [31–33] attribute this observation to the reduced levels of Dicer. However, this is not supported by the findings of Chiosea et al. [34], which showed Dicer upregulation in prostate cancer. Similarly, hypermethylation of promotor regions frequently found in prostate cancer [35] cannot be the sole mechanism underlying the low expression of mature miRNAs in prostate tumor tissues because CpG hypermethylation does not always lead to gene expression downregulation [36]. In this study, we showed a high rate of miRNA editing in prostate tumor compared to non-tumor tissues. The combined number of reads of both edited and wildtype miRNAs in prostate tumor exceeds that found in non-tumor tissues. Our findings suggest that miRNA editing could not only have a significant role in the post-transcriptional regulation of cancer genes but also in the decrease of the wildtype miRNAs observed in prostate tumor tissues.

Ethno-specific genetic variation could affect the prevalence and expression of miRNAs linked to cancer [19, 37–39]. Our findings, showing novel associations between 4 miRNAs and prostate cancer in Arabs, suggest that miRNA expression may contribute to the clinical and pathological diversity and ethnic-related heterogeneity of prostate cancer.

## Conclusions

This study suggests that the identified miRNAs, differentially regulated in prostate cancer, represent putative factors for the increased risk of PCa in Arabs. The role of miRNA editing as a potential mechanism underlying deregulation of cancer genes in prostate cancer can be complemented with other functional analyses. Extension of the findings of the current study to other Arab populations will be of use in determining whether these genetic markers are specific to Arabs.

## Additional files

**Additional file 1: Figure S1.** Average NGS reads length distribution Q:Qatar S: Saudi Arabia N: Non-tumor T: Tumor. **Figure S2.** Scatter Plot of miRNAs in each pair of samples. Each point represents a miRNA. The X axis and Y axis show expression level of miRNAs in tumor and non-tumor tissues respectively. Red points represents miRNAs with ratio > 2; blue points represents miRNAs with 1/2 < ratio ≤2; green points represents miRNAs with ratio ≤1/2. **Table S1.** Patient information. **Table S2.** Primer sequence. **Table S3.** The filtered NGS reads mapped to genome.

**Additional file 2: Table S4.** miRNA reads summary.

**Additional file 3.** miRNA expression comparison in each pair of PCa samples.

**Additional file 4.** miRNA editing summary.

## Abbreviations

PCa: prostate cancer
sRNA: small non-coding RNA
miRNA: microRNA
FFPE: formalin-fixed paraffin-embedded
qPCR: quantitative PCR
KEGG: Kyoto Encyclopedia of Genes and Genomes
PSA: prostate-specific antigen
ncRNA: non-protein coding RNA
NGS: next generation sequencing
bp: base pair
rRNA: ribosomal RNA
snRNA: small nuclear RNAs
snoRNA: small nucleolar RNAs
SOAP: Short Oligonucleotide Analysis Package
scRNA: small cytoplasmic RNA
tRNA: transfer RNA
RT-qPCR: real time-quantitative PCR
KRAS: kirsten rat sarcoma viral oncogene homolog
BCL2: B cell leukemia/lymphoma 2
PTEN: phosphatase and tensin homolog
