# Supplementary material for: Prostate cancer small non-coding RNA transcriptome in Arabs
Source: J Transl Med. 2017 Dec 21;15:260. doi: 10.1186/s12967-017-1362-x (PMC5740966; doi:10.1186/s12967-017-1362-x)
Supplement: Supplementary file 1 — Additional file 1: Figure S1. Average NGS reads length distribution Q:Qatar S: Saudi Arabia N: Non-tumor T: Tumor. Figure S2. Scatter Plot of miRNAs in each pair of samples. Each point represents a miRNA. The X axis and Y axis show expression level of miRNAs in tumor and non-tumor tissues respectively. Red points represents miRNAs with ratio > 2; blue points represents miRNAs with 1/2 < ratio ≤2; green points represents miRNAs with ratio ≤1/2. Table S1. Patient information. Table S2. Primer sequence. Table S3. The filtered NGS reads mapped to genome. [file 12967_2017_1362_MOESM1_ESM.docx]

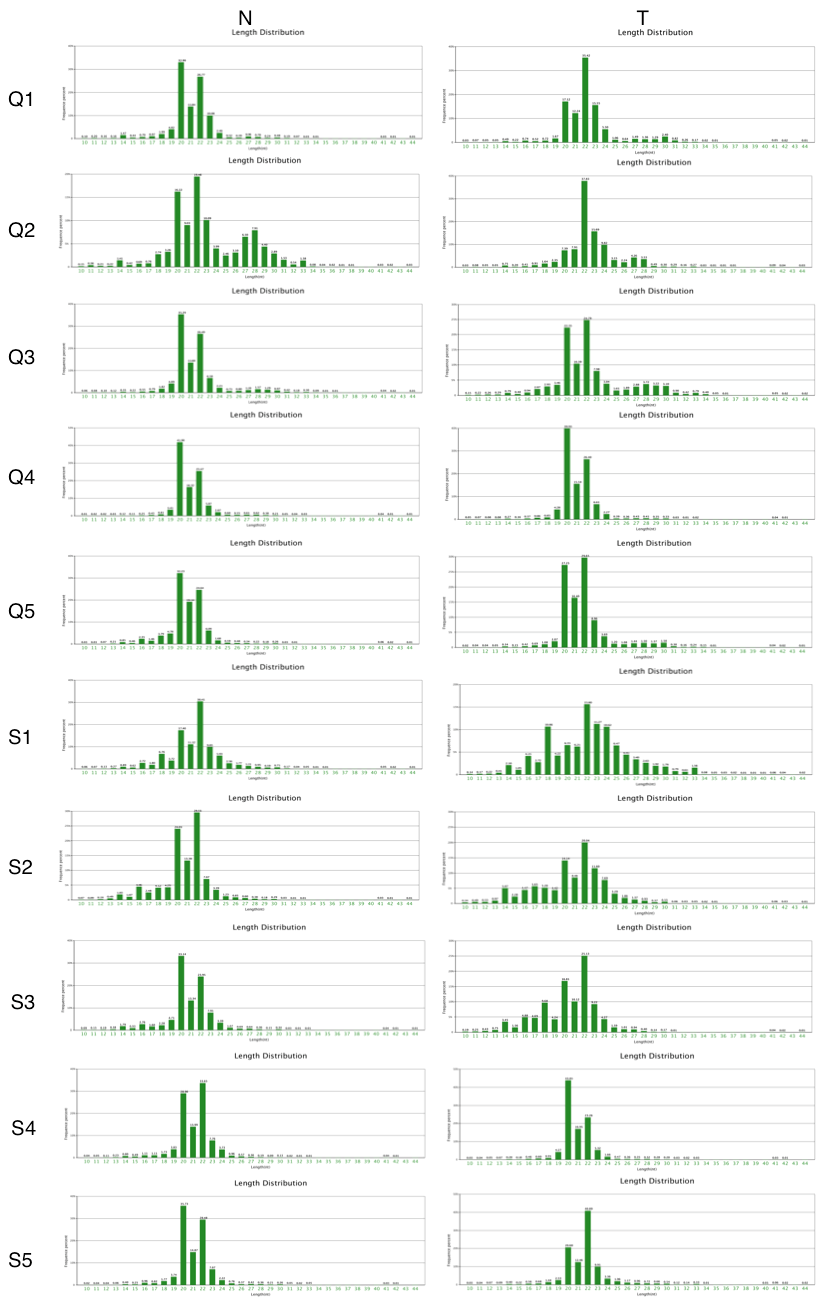


**Fig. S1** Average NGS reads length distribution

Q:Qatar S: Saudi Arabia N: Non-tumor T: Tumor


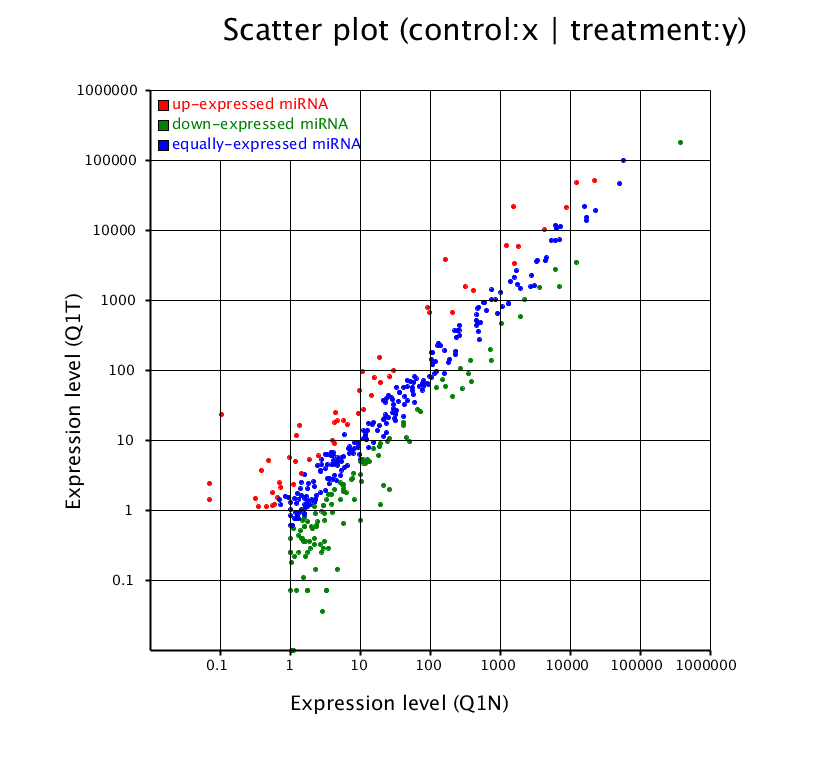

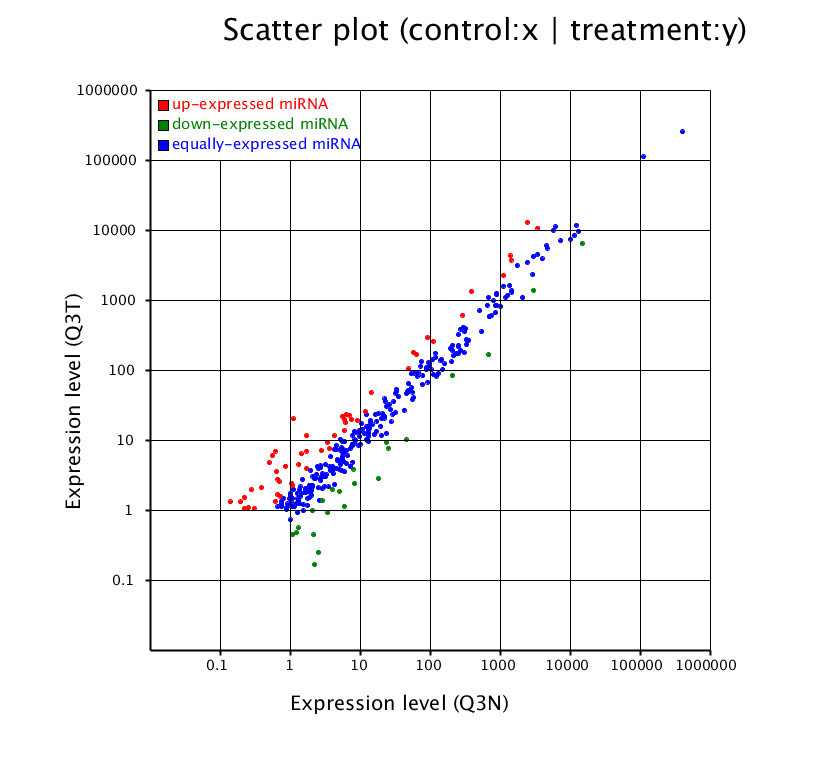

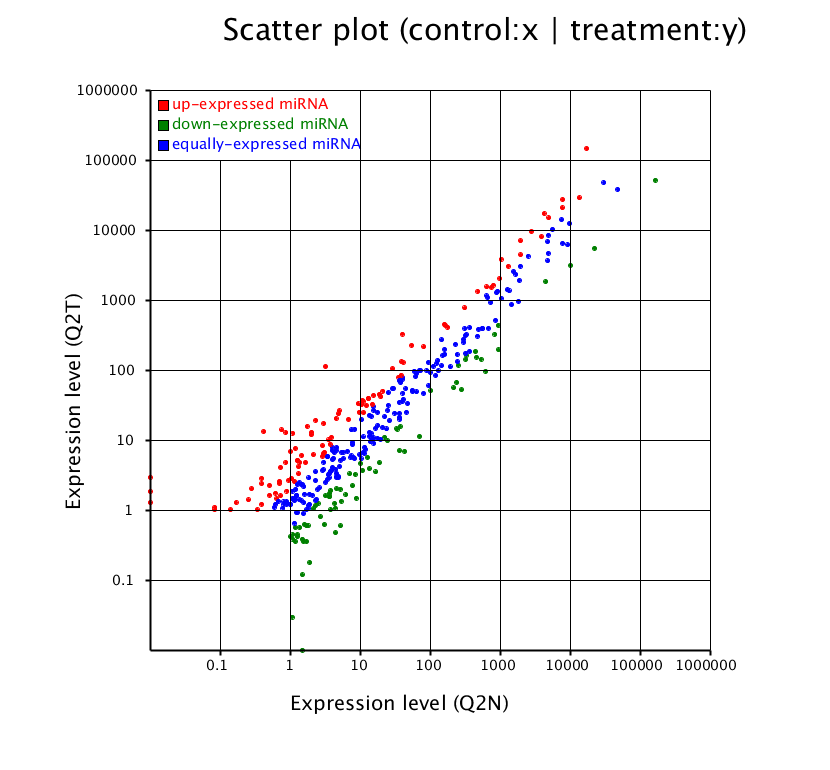

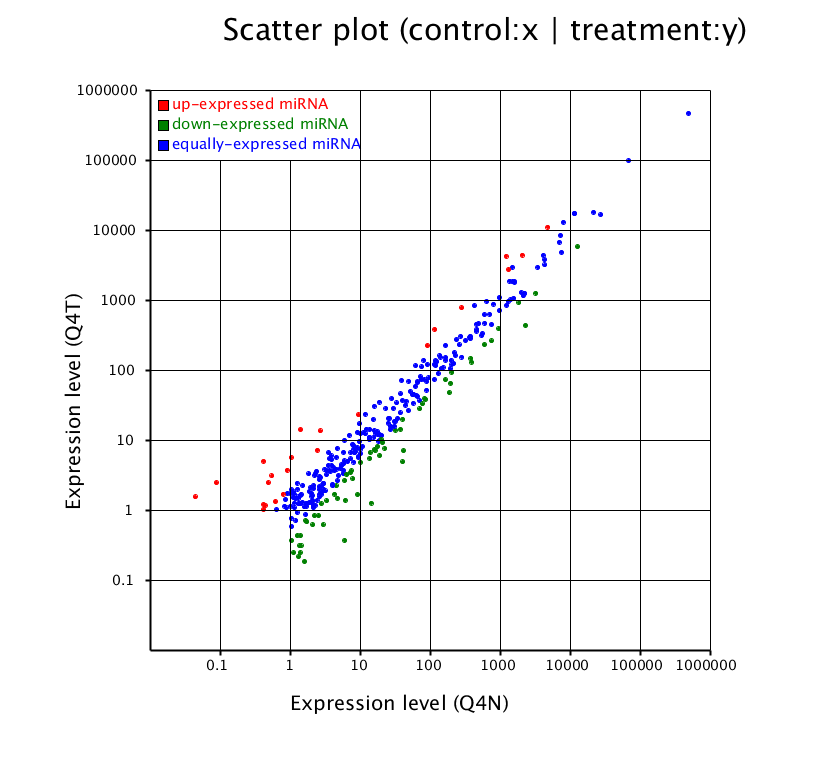

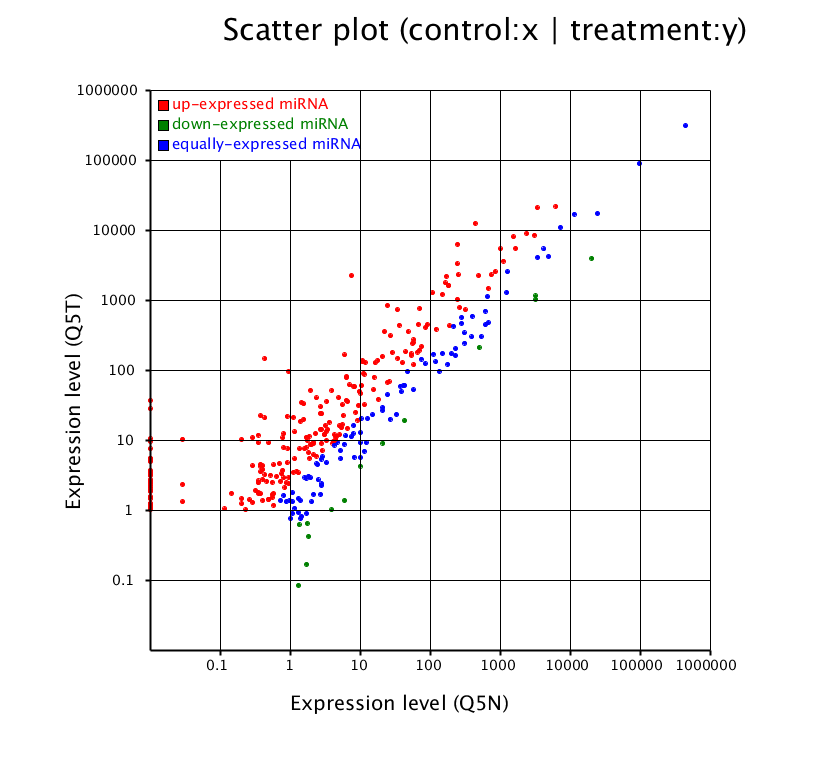

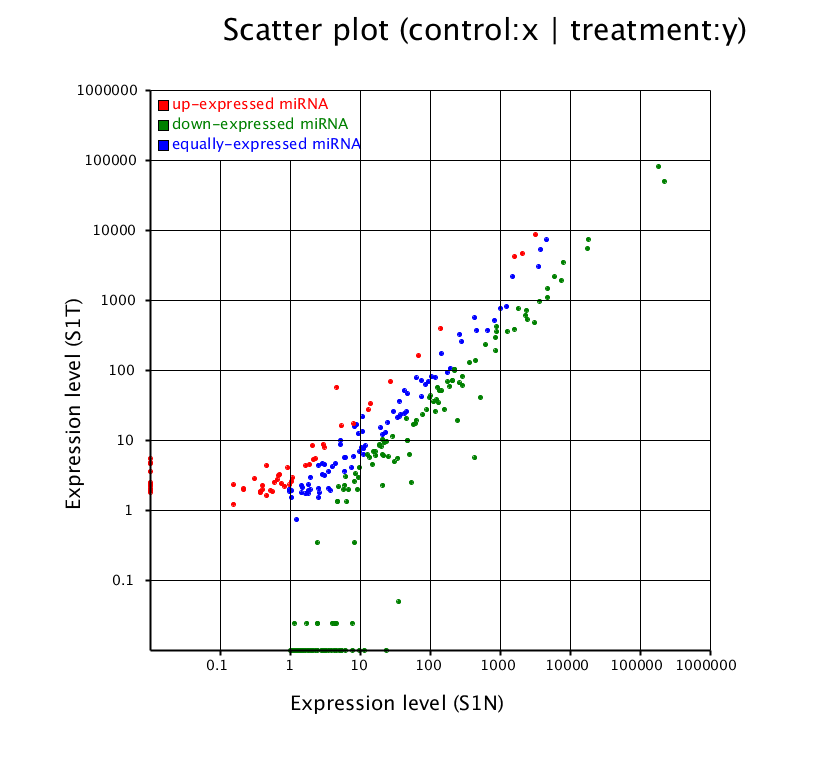

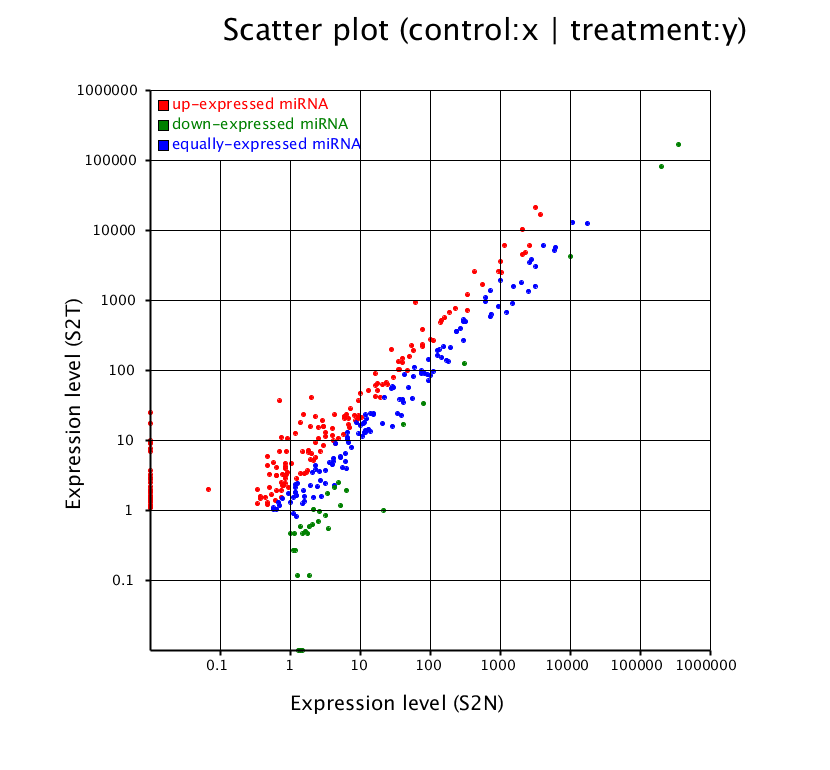

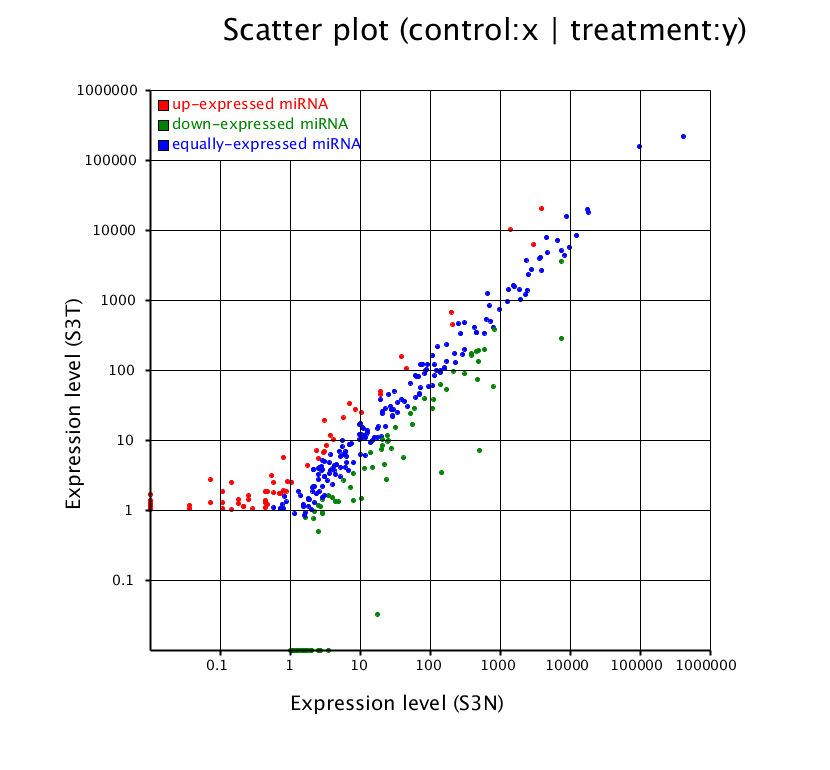

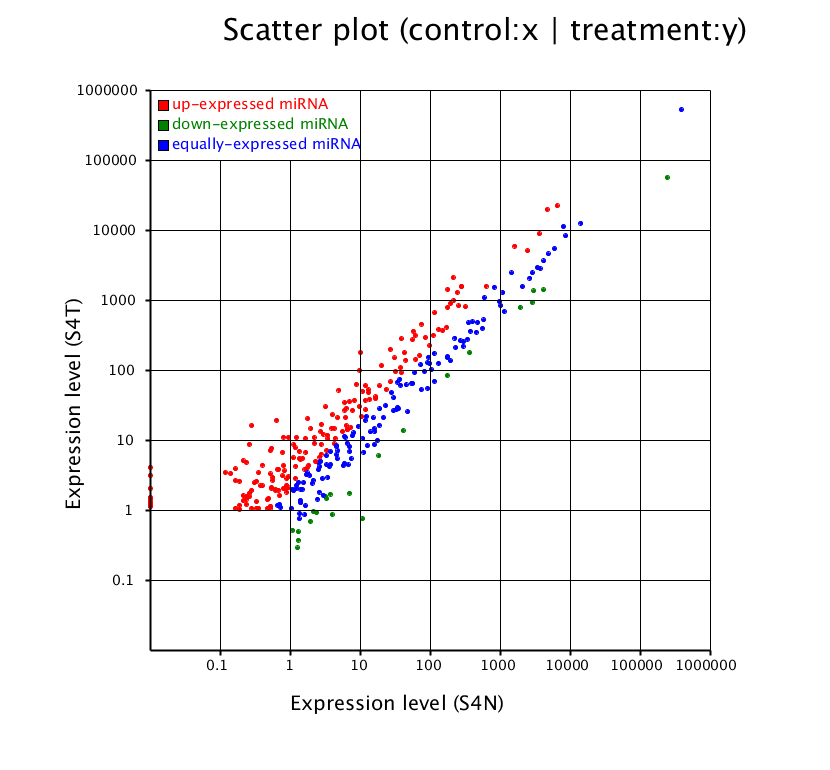

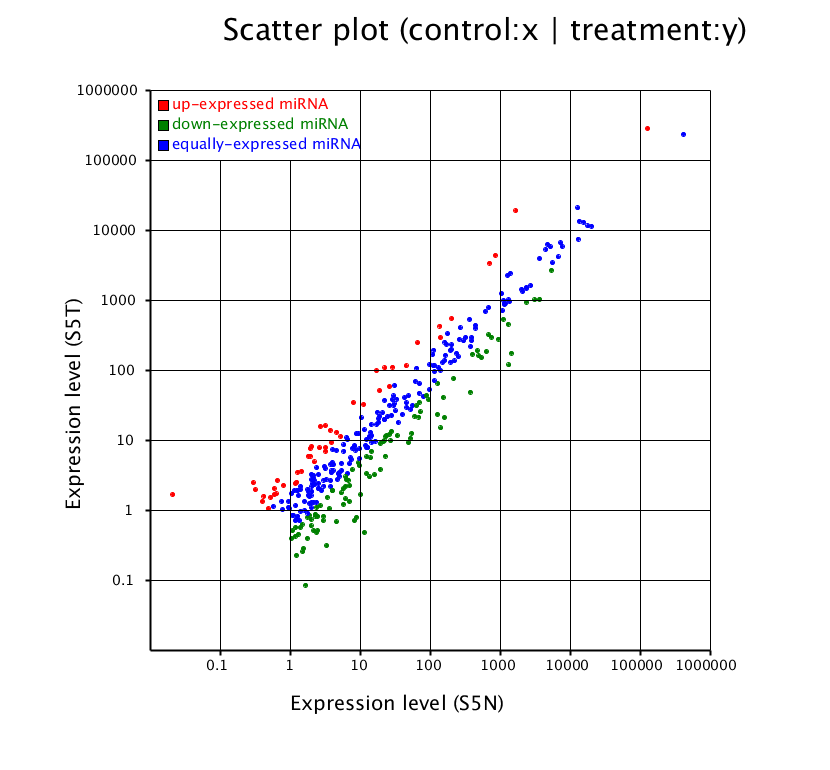


**Fig. S2** Scatter Plot of miRNAs in each pair of samples. Each point represents a miRNA. The X axis and Y axis show expression level of miRNAs in tumor and non-tumor tissues respectively. Red points represents miRNAs with ratio>2; blue points represents miRNAs with 1/2 <ratio<=2; green points represents miRNAs with ratio<=1/2.

**Table S1** Patient information

| Subject | Age | Gleason  Score | Lymphovascular permeation |
| --- | --- | --- | --- |
| Q1 | 66 | 3+3 | 0 |
| Q2 | 68 | 4+3 | 0/1 |
| Q3 | 67 | 3+4 | 0 |
| Q4 | 69 | 3+3 | 0 |
| Q5 | 69 | 3+2 | 0/1 |
| S1 | 82 | 3+4 | 0 |
| S2 | 75 | 3+4 | 0 |
| S3 | 65 | 4+3 | 0 |
| S4 | 72 | 3+3 | 0 |
| S5 | 59 | 3+3 | 0 |

Q: Qatar S: Saudi Arabia

**Table S2** Primer sequence

| Primer | Sequence (5' - 3') |
| --- | --- |
| BCL2-F | TTGCTTTACGTGGCCTGTTTC |
| BCL2-R | GAAGACCCTGAAGGACAGCCAT |
| AKT3-F | AAGGTTGGGTTCAGAAGAGGG |
| AKT3-R | TGAGGGGATAAGGTAAATCCACA |
| KRAS-F | GGGGAGGGCTTTCTTTGTGTA |
| KRAS-R | GTCCTGAGCCTGTTTTGTGTC |
| PTEN-F | GCAGAAAGACTTGAAGGCGTA |
| PTEN-R | TTGGCGGTGTCATAATGTCT |
| P21-F | GACACTGGCCCCTCAAATCG |
| P21-R | CTCCTTGTTCCGCTGCTAATCA |
| MCL1-F | AAGCCAATGGGCAGGTCT |
| MCL1-R | TGTCCAGTTTCCGAAGCAT |

F: Forward R: Reverse

**Table S3** The filtered NGS reads mapped to genome

| Sample | | Total Reads | Mapping to genome | Percentage |
| --- | --- | --- | --- | --- |
| Q1 | N | 28277250 | 22696172 | 80.26% |
|  | T | 27918838 | 21703231 | 77.74% |
| Q2 | N | 35391692 | 27038407 | 76.40% |
|  | T | 33773903 | 26337938 | 77.98% |
| Q3 | N | 35735732 | 27093881 | 75.82% |
|  | T | 36095235 | 26055704 | 72.19% |
| Q4 | N | 45235749 | 35995171 | 79.57% |
|  | T | 32598915 | 25405574 | 77.93% |
| Q5 | N | 34197138 | 25365336 | 74.17% |
|  | T | 35779358 | 25583069 | 71.50% |
| S1 | N | 32055346 | 24309556 | 75.84% |
|  | T | 41112010 | 32879026 | 79.97% |
| S2 | N | 29397073 | 23574522 | 80.19% |
|  | T | 26137643 | 18926233 | 72.41% |
| S3 | N | 27264478 | 21417797 | 78.56% |
|  | T | 30690489 | 24672295 | 80.39% |
| S4 | N | 42147101 | 34880854 | 82.76% |
|  | T | 34881814 | 27122458 | 77.76% |
| S5 | N | 47111742 | 37781855 | 80.20% |
|  | T | 35434376 | 28729997 | 81.08% |

Q: Qatar S: Saudi Arabia N: Non-tumor T: Tumor
